# Two cases of Erythrodermic psoriasis treated with Golimumab

**DOI:** 10.1016/j.amsu.2022.103961

**Published:** 2022-06-08

**Authors:** Mayssoun Kudsi, Mhd Amin Alzabibi, Mosa Shibani

**Affiliations:** a Faculty of Medicine, Syrian Private University, Damascus, Syria; b Faculty of Medicine, Damascus University, Damascus, Syria

**Keywords:** Erythrodermic psoriasis, Golimumab, Biological products, Case series

## Abstract

**Introduction and importance:**

Erythrodermic psoriasis (EP) is a very severe subtype of psoriasis, with a challenge poses in its treatment, as currently available therapies often provide unsatisfactory results, for those many biologics have been used in the treatment of EP such as Golimumab which has been extensively studied for the treatment of psoriatic arthritis, and chronic plaque psoriasis. However, no clinical trials have been performed for EP.

**Case presentation:**

We report two cases of a 23-year old female, and a 31-year male who presented with severe psoriasis that previously un respond to ultraviolet B phototherapy, methotrexate, cyclosporine, and topical agents. Skin lesions worsened progressively and developed into erythroderma. Therefore, we administered golimumab 50 mg, which lead to the improvement of the skin lesions according to the Psoriasis Area and Severity Index score after the first administration; lesions improved further throughout the treatment course.

**Conclusion:**

Golimumab may be an alternative treatment for Erythrodermic psoriasis patients unrespond to other treatments even it did not have the FDA approval, so this is an off label indication and treatment.

## Highlights

• Erythrodermic psoriasis is a very severe subtype of psoriasis, with a challenge poses in its treatment.
• Golimumab is tumor necrosis factor inhibitors.
• Golimumab is FDA approved treatment for psoriatic arthritis, and chronic plaque psoriasis. However, no clinical trials have been performed for the treatment of erythrodermic psoriasis.

## 1 Background

Erythrodermic psoriasis (EP) is considered a rare yet severe variant of psoriasis [1]. EP accounts for approximately 2.5% of all psoriasis cases [2]. It usually results from exacerbation of existing poorly managed psoriasis. However, in some cases it may occur abruptly due to several factors such as reaction to some medication, infection, stress, or sudden withdrawal of systemic medications like corticosteroids. The main clinical presentation in EP is diffuse erythema involving almost all body surface area (75–90%) [1,3]. However, EP patients can also present with non-specific symptoms, such as pruritus, fever, chills, arthralgia, and lymphadenopathy [1].

The pathophysiology of EP still needs further investigation, but some immunological biomarkers are believed to be involved, such as Interleukin-4 (IL-4), Interleukin-10 (IL-10), IgE antibodies, and T-helper-2 lymphocytes [[4], [5], [6], [7]]. The management of EP is challenging for both physicians and patients. Many conventional medications are recommended as first line in treating EP, such as cyclosporine, infliximab, acitretin, and methotrexate [8]. Unfortunately, due to the possible side effects of these drugs, and the patient's dissatisfaction with the results in many cases, the benefit of relying on them in treatment is limited. Recent evidence suggests that biological agents may represent a safer and more reliable choice of medication. Golimumab (GLM), an anti-TNF, immunoglobulin-kappa monoclonal antibody, was approved in both the United States of America and Europe as a treatment for rheumatoid arthritis, Psoriatic Arthritis, and Ankylosing Spondylitis, but not for EP [9]. The recommended dosage was 50 mg monthly as subcutaneous injection [10]. In this case report, we present the first two cases in Syria of EP to be treated with GLM without change in the recommended dosage, to investigate its efficacy in controlling EP.

This case series has been reported in line with the SCARE Criteria [11].

## 2 Cases report

### 2.1 Case 1

**Fig. 1:**
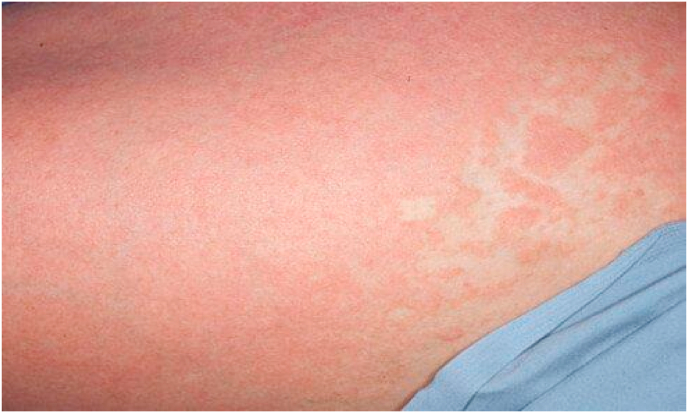
Erythematosus psoriasis, presented in a 23-year-old female.

A 23-year-old female with a 5-year history of severe plaque psoriasis was referred to our clinic in November 2020 for a flare-up of a skin lesion. Physical examination revealed the involvement of 90% of the body surface areas (Fig. 1.), with a Psoriasis Area and Severity Index (PASI) score of 39.1(6). Therefore, her diagnosis was Erythrodermic psoriasis (EP). The patient had no considerable family history nor psychosocial history. conventional treatments were used with poor response including methotrexate (25 mg weekly), acitretin, cyclosporine, topical agent (calcipotriol), and narrow-band ultraviolet B phototherapy. Accordingly, informed consent to undergo off-label biological therapy was obtained, and exclusion of contraindications was performed using laboratory tests including normal complete blood count, urine test, liver, and kidney profile, T-SPOT, HBV test, and lung CT to exclude tuberculosis and hepatitis. The patient received Golimumab 50 mg subcutaneously once every 4 weeks. Great improvement was observed: PASI score decreased 4 weeks after the first injection to 17.1 and reached 11,9 after the second dose (week 8), and 5,4 after the third (week 12) injection. Itching and burning sensations were the only remaining complaint. The patient reserved monthly GLM treatments for 10 months and maintained a reduced PASI score. The treatment was discontinued later due to the Unavailability of the drug.

### 2.2 Case 2

**Fig. 2:**
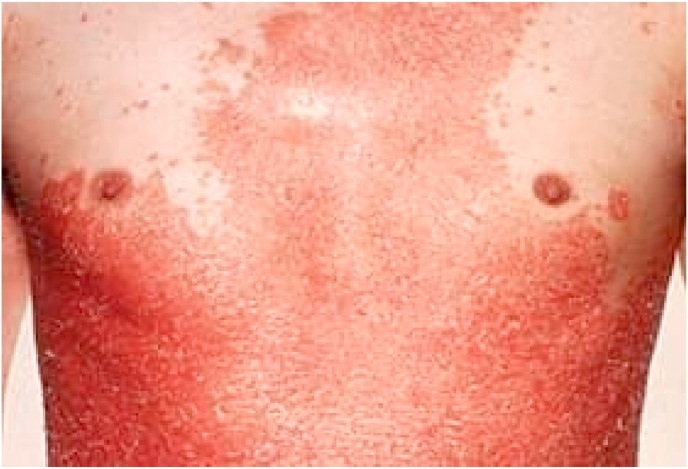
Erythematosus psoriasis, presented in a 31-year-old male.

A 31-year-old male with an 8-year history of plaques psoriasis, who come to the outpatient clinic in January 2021 psoriatic skin involvement (Fig. 2.). His PASI score was 41,9. Therefore, her diagnosis was Erythrodermic psoriasis (EP). The patient had no significant family history nor psychosocial history. The patient was previously treated with conventional treatments including methotrexate (25 mg weekly), cyclosporine, ultraviolet B phototherapy, and topical agents but all treatments were associated with low response. The patient's laboratory results included a normal complete blood count, urine test, liver and kidney profile. To exclude the possibilities of tuberculosis and hepatitis both T-SPOT, HBV test, and lung CT were negative. Due to the severity of the clinical presentation and poor response to conventional systemic therapies, he was considered to have a high need for biologics. Therefore, informed consent to undergo off-label treatment was acquired from the patient, and contraindicated drugs were excluded. The patient received Golimumab 50 mg subcutaneously once every 4 weeks. The patient received great relief from most of the symptoms including rash and swelling. PASI score decreased to 14,8 just 4 weeks after the first injection, 7.6 after the second (week 8), and 4,9 after the third (week 12) injections. The patients who underwent monthly GLM treatments for 8 months maintained a reduced PASI score. The treatment was discontinued due to the lack of the drug.

Both of the previous cases were treated by dermatologists in the Clinic's Department at Al Mouwasat Hospital. And the treatment was given by the dermatologists in hospital to ensure adherence to the treatment.

## 3 Discussion

EP is a rare variation of psoriasis manifested as generalized erythema of the entire body, edema, pruritus, and massive desquamation [12]. If not treated, serious complications may occur including, sepsis, high output cardiac failure, anemia, malabsorption ending in death [13]. In 2009, the medical board of the National Psoriasis Foundation recommended cyclosporine and infliximab to be first-line therapy to their rapid onset of action. Plus Acitretin, methotrexate but they work more slowly [1]. However, traditional treatments, often have limited efficacy and adverse effects, therefore, alternative strategies are required such as biological treatment. Levin et al. reviewed the efficacy and safety of etanercept, ustekinumab, adalimumab, and infliximab in the treatment of EP [8]. While a recent systematic review found a higher level of evidence for infliximab, ixekizumab, ustekinumab, and guselkumab [14]. GLM is FDA approved treatment of psoriatic arthritis [15]. But only one case of 32 years old male in the medical literature to our knowledge described the use of Golimumab in the treatment of EP [16]. In which the patient received GLM 50 mg subcutaneously once per month. This led to a decrease in the patient's PASI from 40.8 to 7.5 after the third dose, In comparison to our two cases, where our patients' PASI improved from 39.1 to 41,9 to 5,4 and 4.9 at the third dose, respectively.

Golimumab is a human monoclonal antibody, used as an immunosuppressive medication that targets tumor necrosis factor-alpha (TNF-α) [15]. TNF-α plays a great role in inflammation as it is functionally known to trigger a series of various inflammatory molecules, including other cytokines and chemokines. Promoting the proinflammatory cytokine cascade, which leads to the recruitment of leukocytes to the lesional epidermis [17]. Furthermore, TNF-α is distributed throughout the epidermis of lesional psoriatic skin and localized to the upper dermal blood vessels [18].

We chose GLM as it was recently demonstrated to exhibit sustained efficacy against multiple facets of psoriatic arthritis including enthesitis and dactylitis.

Our study is considered the second after Lee W–K case report, which shows great clinical improvement in two EP patients when treated with golimumab [16]. patient agreed to be enrolled in this treatment after they suffer from the disease and the use of many treatments. Unfortunately, due to a lack of funding, the treatment was discontinued and the patients went back to the conventional treatments that was given by their original dermatologist and eventually last follow up with the dermatology clinic in Al Mouwasat Hospital.

Further studies are required, although our patients' global assessment and compliance were consistently good, to clarify the long-term profile of GLM for this indication, the rapidity of clearance and excellent safety suggest that Golimumab can play a role in the management of psoriatic arthritis with EP.

## Ethical approval

The ethical approval was given by the Al Mouwasat Hospital IRB committee.

## Source of funding

There is no any funding source.

## Authors' contributions

Mayssoun Kudsi: contributed in writing manuscript and data collecting., Mhd Amin Alzabibi: contributed in writing manuscript., Mosa Shibani: contributed in writing manuscript.

## Trail registry number

Not applicable.

## Guarantor

Mhd Amin Alzabibi.

## Provenance and peer review

Not commissioned, externally peer-reviewed.

## Declaration of competing interest

All authors declare no conflict of interest.
